# 1,25‐Dihydroxyvitamin D protects against age‐related osteoporosis by a novel VDR‐Ezh2‐p16 signal axis

**DOI:** 10.1111/acel.13095

**Published:** 2019-12-26

**Authors:** Renlei Yang, Jie Chen, Jiao Zhang, Ran Qin, Rong Wang, Yue Qiu, Zhiyuan Mao, David Goltzman, Dengshun Miao

**Affiliations:** ^1^ The Research Center for Aging Affiliated Friendship Plastic Surgery Hospital of Nanjing Medical University Nanjing Medical University Nanjing China; ^2^ State Key Laboratory of Reproductive Medicine The Research Center for Bone and Stem Cells Department of Anatomy, Histology and Embryology Nanjing Medical University Nanjing China; ^3^ Department of Anesthesiology Central South University Changsha China; ^4^ Calcium Research Laboratory McGill University Health Centre and Department of Medicine McGill University Montreal Quebec Canada

**Keywords:** cellular senescence, Ezh2, osteogenesis, osteoporosis, p16, Vitamin D

## Abstract

To determine whether 1,25‐dihydroxyvitamin D (1,25(OH)_2_D) can exert an anti‐osteoporosis role through anti‐aging mechanisms, we analyzed the bone phenotype of mice with 1,25(OH)_2_D deficiency due to deletion of the enzyme, 25‐hydroxyvitamin D 1α‐hydroxylase, while on a rescue diet. 1,25(OH)_2_D deficiency accelerated age‐related bone loss by activating the p16/p19 senescence signaling pathway, inhibiting osteoblastic bone formation, and stimulating osteoclastic bone resorption, osteocyte senescence, and senescence‐associated secretory phenotype (SASP). Supplementation of exogenous 1,25(OH)_2_D_3_ corrected the osteoporotic phenotype caused by 1,25(OH)_2_D deficiency or natural aging by inhibiting the p16/p19 pathway. The proliferation, osteogenic differentiation, and ectopic bone formation of bone marrow mesenchymal stem cells derived from mice with genetically induced deficiency of the vitamin D receptor (VDR) were significantly reduced by mechanisms including increased oxidative stress, DNA damage, and cellular senescence. We also demonstrated that p16 deletion largely rescued the osteoporotic phenotype caused by 1,25(OH)_2_D_3_ deficiency, whereas 1,25(OH)_2_D_3_ could up‐regulate the enzyme Ezh2 via VDR‐mediated transcription thereby enriching H3K27me3 and repressing p16/p19 transcription. Finally, we demonstrated that treatment with 1,25(OH)_2_D_3_ improved the osteogenic defects of human BM‐MSCs caused by repeated passages by stimulating their proliferation and inhibiting their senescence via the VDR‐Ezh2‐p16 axis. The results of this study therefore indicate that 1,25(OH)_2_D_3_ plays a role in preventing age‐related osteoporosis by up‐regulating Ezh2 via VDR‐mediated transcription, increasing H3K27me3 and repressing p16 transcription, thus promoting the proliferation and osteogenesis of BM‐MSCs and inhibiting their senescence, while also stimulating osteoblastic bone formation, and inhibiting osteocyte senescence, SASP, and osteoclastic bone resorption.

## Graphical abstract

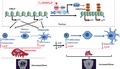

## 1 INTRODUCTION

Vitamin D status influences the overall mineralization of the skeleton, and the rate of bone turnover (Lips & van Schoor, 2011). Epidemiological studies have shown that vitamin D deficiency is a worldwide health problem (Holick, 2007) and could increase the risk of low bone mineral density, osteoporosis, falls, and fractures (Kuchuk, van Schoor, Pluijm, Chines, & Lips, 2009; van Schoor et al., 2008), whereas long‐term supplementation of vitamin D and calcium may be effective in preventing these outcomes (Lips & van Schoor, 2011). Nevertheless, the capacity of calcium and vitamin D supplementation to prevent fractures is still controversial. In a meta‐analysis of 33 randomized clinical trials totaling 51,145 community‐dwelling participants over the age of 50, the use of supplements that included calcium, vitamin D, or both, compared with placebo or no treatment, was not associated with a lower risk of fractures (Zhao, Zeng, Wang, & Liu, 2017). The authors concluded that their findings do not support the routine use of these supplements in community‐dwelling older people. The reasons for this controversy remain unclear. Vitamin D is converted to 25‐hydroxyvitamin D (25OHD) by the action of a liver 25‐hydroxylase and is further metabolized into active 1,25‐dihydroxyvitamin D [1,25(OH)_2_D] by the action of the kidney 25‐hydroxyvitamin D 1α‐hydroxylase (1α(OH)ase) enzyme, encoded by CYP27B1. Active 1,25(OH)_2_D exerts its biological function by binding to the vitamin D receptor (VDR) (Plum & DeLuca, 2010). Production of 1,25(OH)_2_D is influenced by aging and may be reduced by approximately 50% as a result of an age‐related decline in renal function (Gallagher, 2013). Thus, it is possible that the effectiveness of vitamin D supplementation, which is dependent on the effectiveness of 1,25(OH)_2_D production, may as a consequence decline with aging.

It is in this context of multiple aging comorbidities that the gerontology community has increasingly recognized the concept that aging itself is the greatest risk factor for most age‐related chronic diseases, including atherosclerosis, cancers, dementias, diabetes, and osteoporosis (Tchkonia, Zhu, van Deursen, Campisi, & Kirkland, 2013). Some studies have indicated a close association between low levels of vitamin D and these age‐related disorders (Plum & DeLuca, 2010). In the elderly, aging worsens the adverse effects of sex steroid loss on bone by decreasing defenses against oxidative stress, and bone aging is also accompanied by an alteration in the tissue microenvironment with increasing proinflammatory cytokine levels (Farr et al., 2016; McLean, 2009), a critical contributing factor for osteoporosis. In osteoporosis, the amount of bone resorbed is greater than the amount of bone formed leading to a reduction of trabecular and cortical bone volume and density (Chien & Karsenty, 2005; Manolagas & Parfitt, 2010). This decrease in bone mass, as well as a reduction of bone quality, results in decreased bone strength and predisposes the elderly population to an increased risk of fractures. In our previous studies, we found that in mutant mouse models with genetically deleted 1α(OH)ase (1α(OH)ase^−/−^) or VDR (VDR^−/−^), osteoblast numbers, mineral apposition rate, and bone volume were suppressed below levels seen in wild‐type mice (Panda et al., 2004), even when hypocalcemia and secondary hyperparathyroidism were prevented by feeding the animals a high calcium, high phosphorus, lactose‐containing “rescue” diet. This suggested that 1,25(OH)_2_D has a direct bone anabolic action besides its role in maintaining calcium and phosphorus balance. We recently demonstrated that 1,25(OH)_2_D exerts an anti‐aging role by activation of Nrf2‐antioxidant signaling and inactivation of p16/p53 senescence signaling (Chen et al., 2019); however, it is unclear whether 1,25(OH)_2_D plays a role in protection against osteoporosis through its anti‐aging mechanisms.

Oxidative stress is an important cause of cellular senescence. Cellular senescence is the process by which a cell enters a permanent cell cycle block, and senescent cells display a senescence‐associated secretory phenotype (SASP) (Coppe et al., 2008). SASP factors which define this phenotype include the production of proinflammatory cytokines, growth factors, chemokines, and matrix remodeling enzymes (Ovadya & Krizhanovsky, 2014). Senescent cells cause or aggravate the development of aging‐related diseases through their growth arrest phenotype and SASP factors. The cell cycle‐dependent kinase inhibitor p16 is not only a recognized indicator of cellular senescence, but it also acts as a key effector of cellular senescence (Lopez‐Otin, Blasco, Partridge, Serrano, & Kroemer, 2013). During the development of physiological aging and aging‐related diseases, the expression level of p16 is gradually increased (Krishnamurthy et al., 2004). Recent studies have shown that p16‐positive cells in different tissues contribute to the development and progression of aging‐related lesions, resulting in a shortened healthy lifespan, while the elimination of p16‐positive senescent cells can delay the development and progression of senescence‐related lesions in different tissues. Deletion of p16‐positive senescent cells not only prolongs the lifespan of premature aging mice, but also prolongs the lifespan of natural aging mice (Baker et al., 2011, 2016). However, it remains unknown whether p16 deletion can inhibit osteogenic cell senescence by promoting osteogenic cell proliferation and differentiation into osteoblasts.

To determine whether 1,25(OH)_2_D exerts anti‐osteoporosis action by blocking the p16‐cell senescence pathway, wild‐type and 1α(OH)ase^−/−^ mice were fed a high calcium/phosphorus rescue diet or aged 1α(OH)ase^−/−^ mice or 18‐month‐old aged wild‐type mice were subcutaneously injected with 1,25(OH)_2_D_3_. As well, 1α(OH)ase and p16 double knockout mouse models (1α(OH)ase^−/−^p16^−/−^) were generated. The bone phenotypes of the above groups of animals were analyzed using histopathology, cell biology, and molecular biology. Ezh2 is part of the polycomb repressive complex 2 (PRC2 complex) which is responsible for the trimethylation of H3K27 (histone 3) to generate H3K27me3. H3K27me3 is involved in the repression of many genes involved in development and cell differentiation. Down‐regulation of Ezh2 and H3K27me3 has been associated with increased senescence although Ezh2 reduction may act, in part, by directly increasing DNA damage (Ito, Teo, Evans, Neretti, & Sedivy, 2018). We also therefore determined whether 1,25(OH)_2_D mediates transcriptional up‐regulation of Ezh2‐H3K27me3 via the VDR and whether this inhibits the p16 cell senescence pathway.

## 2 RESULTS

### 2.1 1,25(OH)_2_D_3_ deficiency accelerates age‐related bone loss

**Figure 1:**
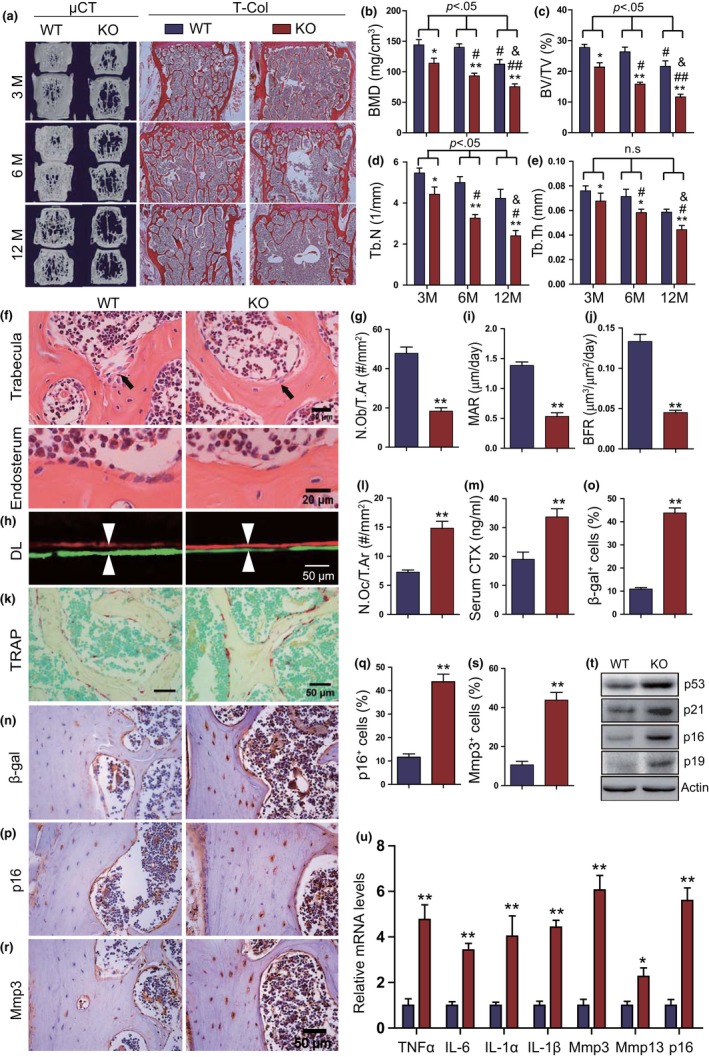
1,25(OH)_2_D_3_ deficiency accelerates aging‐related bone loss. (a) Representative μCT scans of 3D longitudinal reconstructions and total collagen (T‐Col) staining of lumbar vertebrae from 3‐, 6‐, and 12‐month‐old wild‐type (WT) and 1ɑ(OH)ase^−/−^ mice (KO) on a rescue diet (RD). Microtomography indices were measured as (b) bone mineral density (BMD), (c) trabecular bone volume (BV/TV, %), (d) trabecular number (Tb.N), and (e) trabecular thickness (Tb.Th). *, *p* < .05, **, *p* < .01, compared with age‐matched WT mice. #, *p* < .05, ##, *p* < .01, compared with 3‐month‐old genotype‐matched mice. &, *p* < .05, compared with 6‐month‐old genotype‐matched mice. (f) Representative micrographs of vertebral trabecular and cortical sections stained with H&E and (g) a quantitative analysis of the number of osteoblasts per tissue area (N.Ob/T.Ar, #/mm^2^). (h) Representative micrographs of calcein/xylenol orange (XO) dual‐labeling (DL), (i) mineral apposition rate (MAR), and (j) bone formation rate (BFR). (k) Representative micrographs of vertebral trabecular sections stained histochemically for TRAP and (l) a quantitative analysis of the number of osteoclasts per tissue area (N.Oc/T.Ar, #/mm^2^). (m) Serum c‐telopeptide of collagen (CTx) levels (ng/ml). Representative micrographs of vertebral cortical sections immunostained for (n) β‐gal, (p) p16, and (r) matrix metalloproteinase (Mmp) 3. Quantification for the percentages of (o) β‐gal^+^, (q) p16^+^, and (s) Mmp3^+^ osteocytes. (t) Western blots of bone extracts for the expression of p53, p21, p16, and p19. ß‐actin was used as a loading control for Western blots. (u) Lumbar vertebrae with bone marrow removed were subjected to RNA extraction, and the mRNA levels of SASP including TNF‐ɑ, IL‐6, IL‐1ɑ, IL‐1β, Mmp3, Mmp13, and p16 were analyzed using real‐time RT–PCR. *, *p* < .05, **, *p* < .01, ***, *p* < .001, compared with WT mice

To determine whether 1,25(OH)_2_D deficiency accelerated aging‐dependent bone loss, we compared the vertebral phenotype of 3‐, 6‐, and 12‐month‐old wild‐type and 1,25(OH)_2_D deficient [1α(OH)ase^−/−^] mice fed a high calcium/phosphate (rescue) diet using µCT and histochemical staining for total collagen. The rescue diet normalized serum calcium, phosphorus, and PTH levels but did not alter undetectable 1,25(OH)_2_D in 6‐month‐old 1α(OH)ase^−/−^ mice (Figure S1A–D). Bone mineral density (BMD), trabecular bone volume, trabecular number, and thickness were not decreased in 6‐month‐old wild‐type mice compared with 3‐month‐old wild‐type mice; however, BMD and trabecular bone volume were significantly reduced in 12‐month‐old wild‐type mice compared with 3‐month‐old wild‐type mice. By contrast, these parameters decreased progressively in 3‐, 6‐ and 12‐month‐old 1α(OH)ase^−/−^ mice on a rescue diet and were dramatically decreased at each age compared with age‐matched wild‐type mice (Figure 1a–e).

To assess whether aging‐dependent bone loss induced by 1,25(OH)_2_D deficiency was associated with alterations of bone turnover, we analyzed changes of bone formation and resorption in 6‐month‐old wild‐type and 1α(OH)ase^−/−^ mice on the rescue diet by histomorphometric analyses, and calcein and xylenol orange double labeling. We found that the osteoblast number, mineral apposition rate (MAR), bone formation rate (BFR), and long bone strength including maximum load, energy, maximum stress, and elastic modulus were all significantly decreased compared with wild‐type mice (Figure 1f–j, Figure S1E–H). However, TRAP‐positive osteoclast numbers and C‐telopeptide of type 1 collagen (CTX) levels were increased significantly (Figure 1k–m) in 1α(OH)ase^−/−^ mice compared with wild‐type mice.

We then assessed whether aging‐dependent bone loss induced by 1,25(OH)_2_D deficiency was associated with increased osteocyte senescence and senescence‐associated secretory phenotype (SASP); we thus analyzed changes of osteocyte senescence and SASP in 6‐month‐old wild‐type and 1α(OH)ase^−/−^ mice on the rescue diet by immunohistochemical staining, Western blots, and real‐time RT–PCR. The results showed that the percentages of β‐gal^+^, p16^+^ and Mmp3^+^ osteocytes, the protein expression levels of p16, p19, p21, and p53, and the mRNA expression levels of TNFα, IL‐6, IL‐1α, IL‐1β, Mmp3, Mmp13, and p16 were all increased significantly in 1α(OH)ase^−/−^ mice compared with wild‐type mice (Figure 1n–u). These results demonstrated that 1,25(OH)_2_D deficiency accelerates age‐related bone loss by reducing osteoblastic bone formation, increasing osteoclastic bone resorption, increasing p16, and inducing osteocyte senescence and SASP.

### 2.2 Supplementation of exogenous 1,25(OH)_2_D_3_ rescues bone loss induced by 1,25(OH)_2_D deficiency

**Figure 2:**
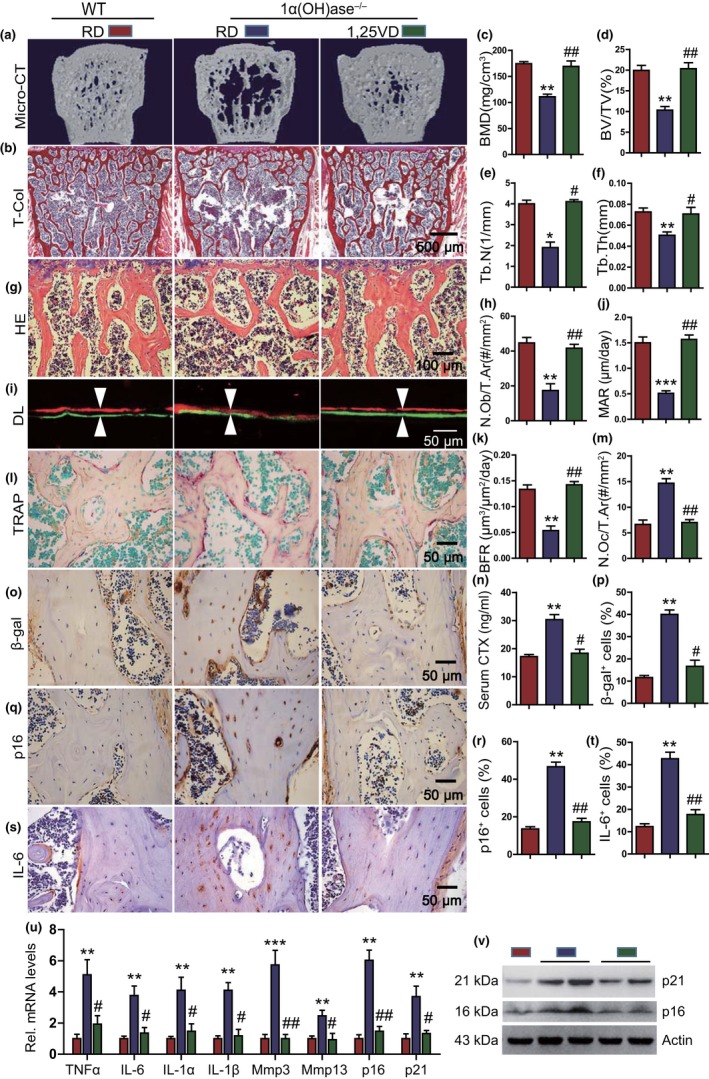
Supplementation of 1,25(OH)_2_D_3_ significantly rescued aging‐related bone loss caused by 1,25(OH)_2_D deficiency. (a) Representative μCT images of lumbar vertebrae of 6‐month‐old WT mice on the rescue diet (RD), 1ɑ(OH)ase^−/−^ mice on the RD or treated with 1,25(OH)_2_D_3_. (b) Representative micrographs of vertebral sections stained for total collagen (T‐Col). Microtomography indices were measured as (c) bone mineral density (BMD), (d) trabecular bone volume (BV/TV, %), (e) trabecular number (Tb.N), and (f) trabecular thickness (Tb.Th). (g) Representative micrographs of vertebral sections stained with H&E and (h) a quantitative analysis of the number of osteoblasts per tissue area (N.Ob/T.Ar, #/mm^2^). (i) Representative micrographs of calcein/xylenol orange (XO) dual‐labeling, (j) MAR, and (k) BFR. (l) Representative micrographs of vertebral trabecular sections stained histochemically for TRAP and (m) a quantitative analysis of the number of osteoclasts per tissue area (N.Oc/T.Ar, #/mm^2^). (n) Serum CTx levels (ng/ml). Representative micrographs of vertebral cortical sections immunostained for (o) β‐gal, (q) p16, and (s) IL‐6. Quantification for the percentages of (p) β‐gal^+^, (r) p16^+^, and (t) IL‐6^+^ osteocytes. (u) Lumbar vertebrae with bone marrow removed were subjected to RNA extraction, and the mRNA levels of SASP including TNF‐ɑ, IL‐6, IL‐1ɑ, IL‐1β, Mmp3, Mmp13, p16, and p19 were analyzed using real‐time RT–PCR. (v) Western blots of bone extracts for the expression of p16 and p21. ß‐actin was used as loading control for Western blots. *, *p* < .05, **, *p* < .01, ***, *p* < .001, compared with WT; #, *p* < .05, ##, *p* < .01, ###, *p* < .001, compared with 1ɑ(OH)ase^−/−^ mice

To assess whether supplementation of exogenous 1,25(OH)_2_D_3_ can prevent bone loss caused by 1,25(OH)_2_D deficiency, 1α(OH)ase^−/−^ mice were either fed the rescue diet after weaning or were injected with 1,25(OH)_2_D_3_ subcutaneously (1 μg/kg, thrice/week); wild‐type mice were fed the rescue diet as the control. Vertebral phenotypes were then analyzed in 6‐month‐old mice. Results showed that both the rescue diet and exogenous 1,25(OH)_2_D_3_ supplementation normalized serum calcium and phosphorus in 6‐month‐old 1α(OH)ase^−/−^ mice (Figure S1A, B). However, in 1α(OH)ase^−/−^ mice supplemented with exogenous 1,25(OH)_2_D_3_ compared with 1α(OH)ase^−/−^ mice on the rescue diet, the BMD, trabecular bone volume, trabecular number and thickness, osteoblast number, MAR, and BFR were all increased significantly (Figure 2a–k), whereas TRAP‐positive osteoclast number, serum CTX levels, the percentages of β‐gal^+^, p16^+^, and IL‐6^+^ osteocytes, the mRNA levels of TNFα, IL‐6, IL‐1α, IL‐1β, Mmp3, Mmp13, p16, and p21, and the protein expression levels of p16 and p21 were all significantly decreased (Figure 2l–v). Most parameters in the 1α(OH)ase^−/−^ mice supplemented with exogenous 1,25(OH)_2_D_3_ were comparable to those of wild‐type mice. These results demonstrated that supplementation with exogenous 1,25(OH)_2_D_3_ rescued bone loss induced by 1,25(OH)_2_D_3_ deficiency by increasing osteoblastic bone formation, inhibiting osteoclastic bone resorption, osteocyte senescence, and SASP.

### 2.3 Supplementation of exogenous 1,25(OH)_2_D_3_ prevents bone loss induced by natural aging

**Figure 3:**
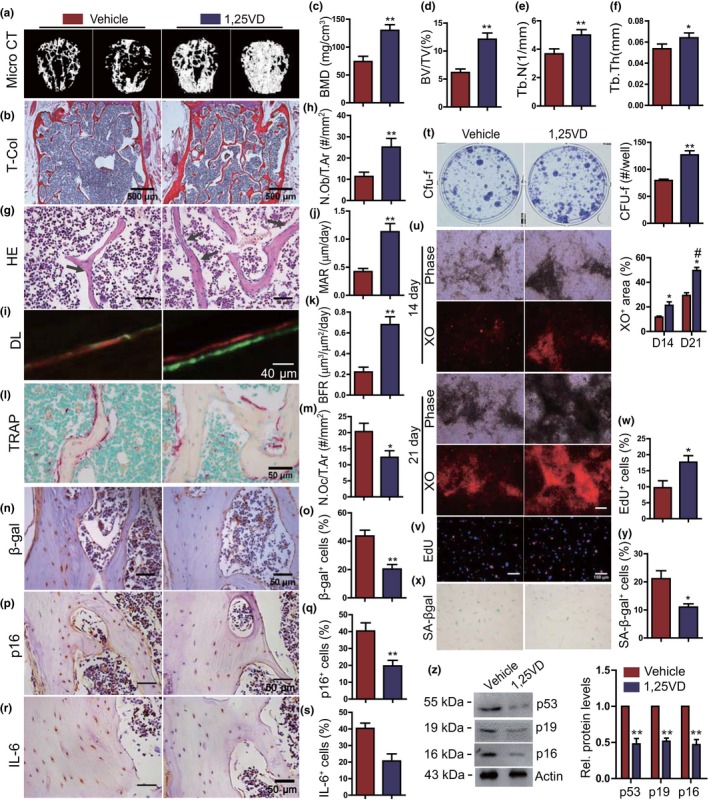
Supplementation of exogenous 1,25(OH)_2_D_3_ prevents bone aging induced by natural aging. (a) Representative μCT images of lumbar vertebrae of 18‐month‐old WT mice which received thrice weekly subcutaneous injections of 1,25(OH)_2_D_3_ at a dose of 0.1 μg/kg or vehicle for 6 months. (b) Representative micrographs of vertebral sections stained for total collagen (T‐Col). Microtomography indices were measured as (c) bone mineral density (BMD), (d) trabecular bone volume (BV/TV, %), (e) trabecular number (Tb.N), and (f) trabecular thickness (Tb.Th). (g) Representative micrographs of vertebral sections stained with H&E and (h) a quantitative analysis of the number of osteoblasts per tissue area (N.Ob/T.Ar, #/mm^2^). (i) Representative micrographs of calcein/xylenol orange (XO) dual‐labeling, (j) MAR, and (k) BFR. (l) Representative micrographs of vertebral trabecular sections stained histochemically for TRAP and (m) a quantitative analysis of the number of osteoclasts per tissue area (N.Oc/T.Ar, #/mm^2^). Representative micrographs of vertebral cortical sections immunostained for (n) β‐gal, (p) p16, and (r) IL‐6. Quantification for the percentages of (o) β‐gal^+^, (q) p16^+^, and (s) IL‐6^+^ osteocytes. Ex vivo primary bone marrow cells cultured for 14 days from vehicle or 1,25(OH)_2_D_3_‐treated 18‐month‐old WT mice and stained with (t) methylene blue to show total CFU‐f and a quantitative analysis of CFU‐f numbers per well, or (u) cultured for 14 or 21 days and stained with xylenol orange (XO) followed by a quantitative analysis for the percentage of XO^+^ cells, or (v) cultured for 14 day for 5‐ethynyl‐2′‐deoxyuridine (EdU) incorporation (cell proliferation) or (x) stained cytochemically for senescence‐associated β‐gal (SA‐β‐gal); quantitative analysis for the percentage of (w) EdU^+^ cells and (y) SA‐β‐gal^+^ cells. (z) Western blots of bone extracts for the expression of p53, p19, and p16. ß‐actin was used as loading control for Western blots. *, *p* < .05, **, *p* < .01, ***, *p* < .001, compared with vehicle

We compared the percentage of senescent osteocytes and the osteogenic capacity of 3‐ and 18‐month‐old wild‐type mice and found that the percentages of β‐gal^+^ and p16^+^ osteocytes were increased from <10% in 3‐month‐old mice to >40% in 18‐month‐old mice, comparable to the levels of 6‐month‐old 1α(OH)ase^−/−^ mice (Figure S2A, B). We also found that xylenol orange (XO)‐positive calcified nodules forming in cultures of bone marrow mesenchymal stem cells (BM‐MSCs) were reduced significantly in 18‐month‐old mice compared with 3‐month‐old mice (Figure S2C, D). To determine whether supplementation of exogenous 1,25(OH)_2_D_3_ could prevent bone aging induced by natural aging, 12‐month‐old wild‐type mice were injected with 1,25(OH)_2_D_3_ subcutaneously (0.1 μg/kg, thrice/week) or vehicle for 6 months, and their vertebral phenotypes were analyzed at 18 months of age. Serum calcium and phosphorus levels were increased significantly in exogenous 1,25(OH)_2_D_3_‐supplemented 18‐month‐old mice compared with vehicle‐treated mice (Figure S2E, F). In addition, BMD, trabecular bone volume, trabecular number and thickness, osteoblast number, MAR, and BFR were increased significantly (Figure 3a–k), whereas TRAP‐positive osteoclastic number, the percentages of β‐gal^+^, p16^+^, and IL‐6^+^ osteocytes, and the mRNA levels of TNFα, IL‐1α, IL‐1β, IL‐6, Mmp3, and p16 were all significantly decreased (Figure 3l–s, Figure S2G) in exogenous 1,25(OH)_2_D_3_‐supplemented 18‐month‐old mice compared with vehicle‐treated mice. The elevations in serum calcium and phosphorus levels may therefore have resulted from increased intestinal absorption rather than bone resorption. We then examined the proliferation, osteogenic differentiation, and senescence of BM‐MSCs derived from vehicle‐ or 1,25(OH)_2_D_3_‐treated 18‐month‐old mice ex vivo. The results revealed that CFU‐f forming efficiency, and the percentages of XO‐positive area and EdU‐positive cells were significantly increased (Figure 3t–w), whereas the percentage of SA‐β‐gal‐positive cells and protein expression levels of p16, p19, and p53 were reduced significantly (Figure 3x–z) in ex vivo cultured BM‐MSCs from 1,25(OH)_2_D_3_‐treated 18‐month‐old mice compared with those from vehicle‐treated mice. These results demonstrated that supplementation of exogenous 1,25(OH)_2_D_3_ could prevent bone loss induced by natural aging through stimulating proliferation and osteogenic differentiation and osteoblastic bone formation, and inhibiting senescence of BM‐MSCs and osteocytes.

### 2.4 VDR deficiency induces BM‐MSC senescence and inhibits their osteogenesis

**Figure 4:**
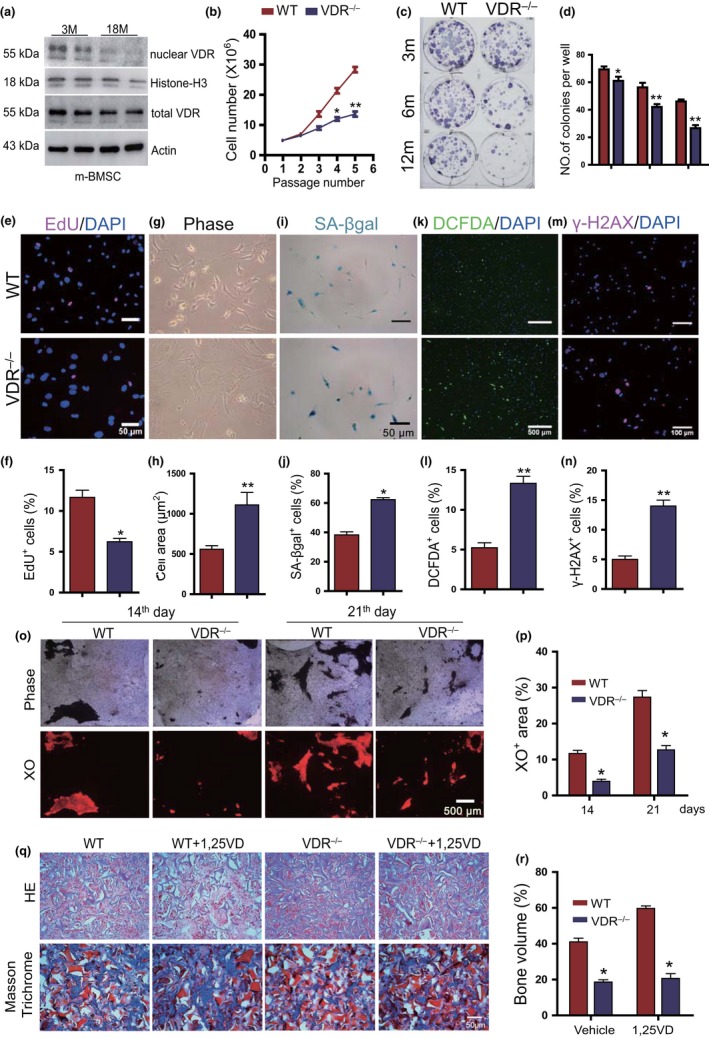
VDR deficiency induces BM‐MSC senescence and inhibits osteogenesis. (a) Western blot of BM‐MSC extracts from young (3 months) and old (18 months) mice for expression of nuclear VDR and total VDR. Histone‐H3 was used as nucleoprotein loading control, whereas ß‐actin was used as total protein loading control for Western blots. (b) In vitro population doublings of BM‐MSCs from 6‐month‐old WT and VDR^−/−^ mice. (c) Ex vivo primary bone marrow cultures from 3‐, 6‐ and 12‐month‐old WT mice stained with methylene blue to show total CFU‐f. (d) Quantification of the number of CFU‐F colonies. Representative micrographs of the second passaged BM‐MSCs from 6‐month‐old WT and VDR^−/−^ mice (e) stained immunocytochemically for EdU, and (g) phase images, or (i) stained cytochemically for SA‐β‐gal to detect senescence, (k) with 2',7'‐dichlorofluorescin diacetate (DCFDA) for reactive oxygen species (ROS) and (m) stained immunocytochemically for γ‐H2AX as a marker of DNA damage. Quantification for (f) the percentages of EdU^+^ cells, (h) average cell area, the percentages of (j) SA‐β‐gal^+^, (l) DCFDA^+^, and (n) γ‐H2AX^+^ cells. (o) Representative micrographs of BM‐MSC cultures under osteogenic differentiation medium from 6‐month‐old WT and VDR^−/−^ mice for 14 or 21 days stained with xylenol orange (XO) and (p) a quantitative analysis for the percentage of XO^+^ cells. (q) The BM‐MSCs from WT and VDR^−/−^ mice were cultured with vehicle or 10^−8^M 1,25(OH)_2_D_3_ and were subcutaneously transplanted; after 6 weeks, transplants were harvested and stained with H&E (upper part of panel q) or Masson trichrome (bottom of panel q). (r) The analysis of bone volume based on H＆E staining. *, *p* < .05, **, *p* < .01, compared with WT mice

We next assessed whether 1,25(OH)_2_D acts via the VDR in producing senescence in BM‐MSCs. First, the nucleoprotein and total protein expression levels of VDR were compared in BM‐MSCs derived from 3‐ and 18‐month‐old wild‐type mice. Although total protein expression levels of VDR did not decrease significantly in BM‐MSCs from 18‐month‐old wild‐type mice compared with those from 3‐month‐old wild‐type mice, the nucleoprotein expression levels of VDR were markedly reduced (Figure 4a). We then compared the proliferation and senescence of BM‐MSCs derived from 6‐month‐old wild‐type and VDR^−/−^ mice. Proliferation capacity, including population doubling, number of CFU‐f, and percentage of EdU^+^ cells were significantly decreased (Figure 4b–f), whereas cell sizes and the percentages of SA‐βgal^+^, DCFDA^+^, and γ‐H2AX^+^ cells were significantly increased in the second passaged VDR^−/−^ BM‐MSCs compared to the second passaged wild‐type BM‐MSCs (Figure 4g–n). Moreover, VDR^−/−^ BM‐MSCs showed reduced osteogenic potential as evidenced by decreased XO^+^ area stained at 14 and 21 days following osteogenic induction (Figure 4o, p). To assess whether 1,25(OH)_2_D_3_ stimulated osteogenesis in vivo via VDR, wild‐type and VDR^−/−^ BM‐MSCs were treated with vehicle or 10^−8^M 1,25(OH)_2_D_3_, respectively, prior to being implanted subcutaneously into recipient SCID mice, and implants were analyzed by staining with H&E and Masson trichrome stain. Ectopic bone volume was significantly reduced in VDR^−/−^ BM‐MSCs compared with wild‐type BM‐MSCs; however, it was markedly increased in 1,25(OH)_2_D_3_‐treated wild‐type BM‐MSCs, but not in 1,25(OH)_2_D_3_‐treated VDR^−/−^ BM‐MSCs (Figure 4q, r). These results suggest that 1,25(OH)_2_D_3_, acting via the VDR, stimulated osteogenesis by promoting proliferation, and inhibiting oxidative stress, DNA damage, and senescence of BM‐MSCs.

### 2.5 1,25(OH)_2_D_3_ inhibits BM‐MSC senescence by VDR‐mediated transcriptional up‐regulation of Ezh2 and repression of p16/p19

**Figure 5:**
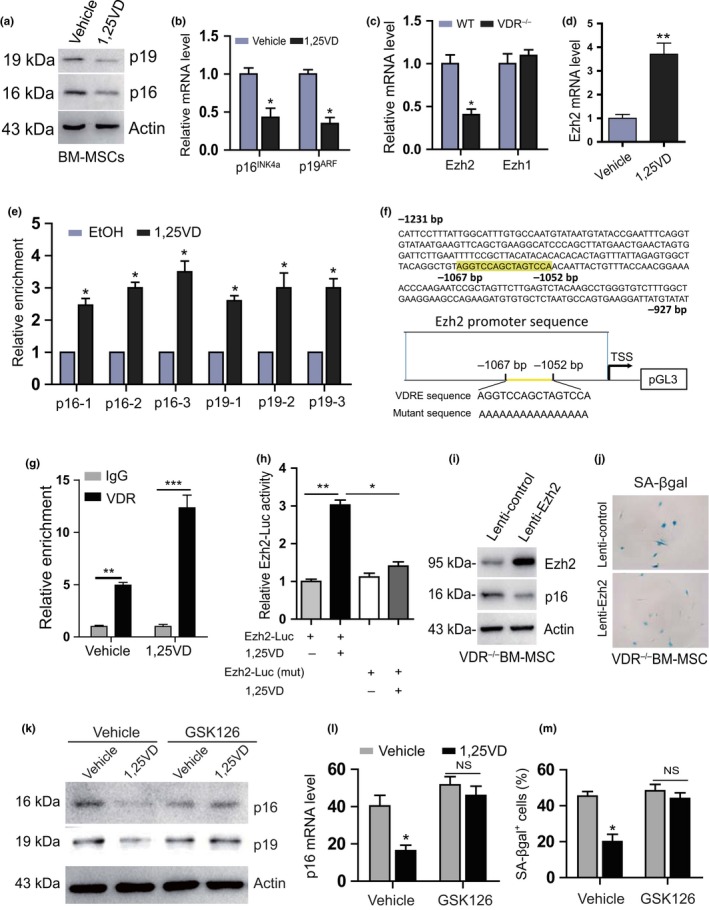
1,25(OH)_2_D_3_ inhibits BM‐MSC senescence by VDR‐mediated transcriptional up‐regulation of Ezh2 and repression of p16/p19. (a) Western blots of the second passaged BM‐MSCs extracts from cultures treated with vehicle or 1,25(OH)_2_D_3_ for 3 days for the expression of p19 and p16. ß‐actin was used as loading control for Western blots. (b) p16 and p19 relative expression levels. (c) The Ezh2 and Ezh1 mRNA relative expression levels of in WT and VDR^−/−^ BM‐MSCs. (d) The Ezh2 mRNA‐related expression levels following 1,25(OH)_2_D_3_ treatment for 12 hr. (e) Chromatin immunoprecipitation (ChIP)‐qPCR assays with H3K27me3 antibody or IgG antibody were performed using vehicle‐ and 1,25(OH)_2_D_3_‐treated BM‐MSCs. (f) VDR‐like elements in mouse Ezh2 promoter region and the mutated VDRE sequence highlighted in yellow (upper region of panel f); schematic structural diagram of pGL3‐Ezh2 promoter and mutant pGL3‐Ezh2 Luc reporter plasmid (lower region of panel f). (g) Analysis of VDR binding to Ezh2 promoter using ChIP. (h) Mouse Ezh2 promoter or Ezh2 promoter mutant Luc‐plasmid were transfected into BM‐MSCs following vehicle or 1,25(OH)_2_D_3_ treatment for 12 hr, and relative luciferase activity was analyzed after 48 hr. *, *p* < .05, **, *p* < .01, compared with BM‐MSCs treated with vehicle. (i) VDR^−/−^ BM‐MSCs were transfected with lenti‐control or lenti‐Ezh2, and the protein expression levels for Ezh2 and p16 were detected using Western blots. (j) Senescent VDR^−/−^ BM‐MSCs were decreased following Ezh2 overexpression detected by SA‐βgal staining. (k) The protein expression levels of p16 and p19 in BM‐MSCs treated with vehicle or the Ezh2 inhibitor, GSK126, in the presence or absence of 1,25(OH)_2_D_3_. (l) p16 mRNA levels and (m) SA‐βgal^+^ cells in vehicle‐ or GSK126‐treated BM‐MSCs in the presence or absence of 1,25(OH)_2_D_3_. *, *p* < .05, compared with BM‐MSCs treated with vehicle

Ezh2 increases the repressive mark H3K27me3 at the INK4a/ARF locus, which encodes the INK4 family of cyclin‐dependent kinase inhibitors, p15^INK4b^ and p16^INK4a^, and a tumor suppressor p19/p14^ARF^ (Aguilo, Zhou, & Walsh, 2011; Cakouros et al., 2012; Kaneda et al., 2011). To determine whether 1,25(OH)_2_D_3_ inactivated p16/p19 signaling by up‐regulating Ezh2‐H3K27me3, we examined the effect of 1,25(OH)_2_D_3_ on the expression of Ezh2 and p16/p19 in BM‐MSCs in vitro. We found that 1,25(OH)_2_D_3_ increased the mRNA level of Ezh2 and down‐regulated p16 and p19 expression at both protein and mRNA levels (Figure 5a–d). We also examined the status of H3K27me3 along the INK4a/ARF locus in vehicle‐ and 1,25(OH)_2_D_3_‐treated BM‐MSCs using ChIP assays. Significantly enriched H3K27me3 was detected in the regions just upstream of the transcription start site (TSS) (p16‐1, p19‐1), surrounding the TSS (p16‐2, p19‐2), and within the intron immediately downstream of the TSS (p16‐3, p19‐3) of p16^INK4a^/p19^ARF^ in 1,25(OH)_2_D_3_‐treated BM‐MSCs compared with vehicle‐treated BM‐MSCs (Figure 5e). To further determine whether 1,25(OH)_2_D_3_ regulates Ezh2 via the VDR at a transcriptional level, a VDRE‐like sequence at 2 kb upstream of the *Ezh2* gene (http://jaspar.genereg.net/) was identified (Figure 5f). The results of a ChIP‐PCR assay demonstrated that the VDR could directly bind to the *Ezh2* promoter at the predicted binding site (Figure 5g). Luciferase reporter assays demonstrated that treatment of 1,25(OH)_2_D_3_ increased luciferase activity significantly in BM‐MSCs transfected with an Ezh2‐Luc plasmid, but failed to activate the mutant Luc reporter (Figure 5h). To assess whether Ezh2 is an effector of the action of VDR to prevent BM‐MSC senescence, the second passaged BM‐MSCs derived from VDR^−/−^ mice were transduced with lentivirus‐Ezh2 or lentivirus empty vector. The results showed that Ezh2 overexpression in VDR^−/−^ BM‐MSCs significantly down‐regulated the protein expression levels of p16 (Figure 5i) and reduced the number of SA‐βgal^+^ cells (Figure 5j). Moreover, the reduced mRNA and protein levels of p16 and p19 and diminished SA‐βgal^+^ cells induced by 1,25(OH)_2_D_3_ treatment were partly blocked following treatment with the Ezh2 inhibitor, GSK126 (Figure 5k–m). These results suggest that 1,25(OH)_2_D_3_ inhibits BM‐MSC senescence by VDR‐mediated transcriptional up‐regulation of Ezh2 and repression of p16/p19.

### 2.6 Deletion of p16 largely rescues bone aging phenotypes induced by 1,25(OH)_2_D deficiency

**Figure 6:**
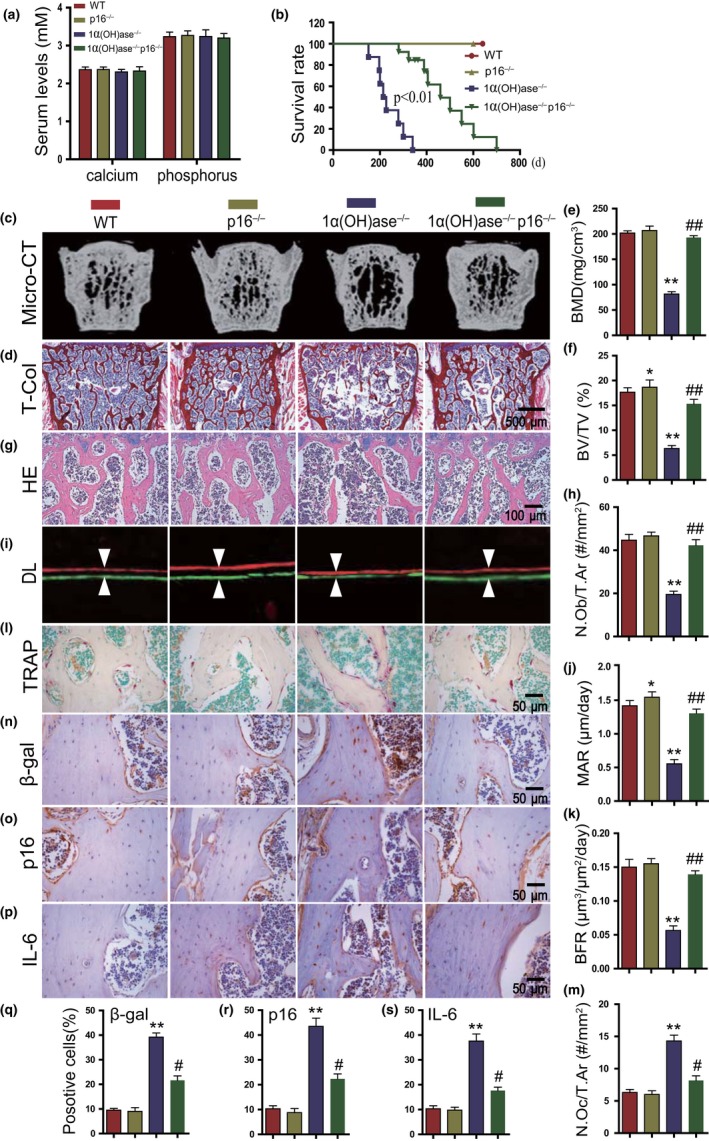
Deletion of p16 largely rescues bone aging phenotypes induced by 1,25(OH)_2_D deficiency. (a) Serum calcium and phosphorus levels and (b) survival rate of WT, p16^−/−^, 1ɑ(OH)ase^−/−^, and 1ɑ(OH)ase^−/−^p16^−/−^ mice on the RD. (c) Representative μCT images of lumbar vertebrae of genetically modified mice. (d) Representative micrographs of vertebral sections stained for total collagen (T‐Col). (e) Bone mineral density (BMD) and (f) trabecular bone volume (BV/TV, %). (g) Representative micrographs of vertebral sections stained with H&E and (h) a quantitative analysis of the number of osteoblasts per tissue area (N.Ob/T.Ar, #/mm^2^). (i) Representative micrographs of calcein/xylenol orange (XO) dual‐labeling, (j) MAR, and (k) BFR. (l) Representative micrographs of vertebral trabecular sections stained histochemically for TRAP and (m) a quantitative analysis of the number of osteoclasts per tissue area (N.Oc/T.Ar, #/mm^2^). Representative micrographs of vertebral cortical sections immunostained for (n) β‐gal, (o) p16, and (p) IL‐6. Quantification for the percentages of (q) β‐gal^+^, (r) p16^+^, and (s) IL‐6^+^ osteocytes. *, *p* < .05, **, *p* < .01, ***, *p* < .001, compared to WT mice. #, *p* < .05, ##, *p* < .01, compared to 1ɑ(OH)ase^−/−^ mice

We recently reported that p16 deletion can partly postpone aging in 1α(OH)ase^−/−^ mice on a normal diet (Chen et al., 2019); we now assessed whether p16 deletion could also postpone aging and rescue bone aging phenotypes in 1α(OH)ase^−/−^ mice on the rescue diet. Compound mutant mice with homozygous deletion of both p16 and 1α(OH)ase [1α(OH)ase^−/−^p16^−/−^] were generated and fed the rescue diet; their lifespan and bone phenotypes were then compared with 1α(OH)ase^−/−^ on a rescue diet and wild‐type littermates. Serum calcium and phosphate levels 1α(OH)ase^−/−^p16^−/−^ mice were not significantly altered (Figure 6a). The median lifespan was significantly extended from 31 weeks in 1α(OH)ase^−/−^ mice to 65 weeks in 1α(OH)ase^−/−^p16^−/−^ mice (Figure 6b). BMD, trabecular bone volume, trabecular number and thickness, osteoblast number, MAR, and BFR were all significantly increased (Figure 6c–k, Figure S3A,B), whereas TRAP‐positive osteoclast numbers, the percentages of β‐gal^+^, p16^+^, and IL‐6^+^ osteocytes, and the mRNA expression levels of TNFα, IL‐6, IL‐1α, IL‐1β, Mmp3, and Mmp13 were all reduced significantly (Figure 6l–s, Figure S3C) in 6‐month‐old 1α(OH)ase^−/−^p16^−/−^ mice compared with 1α(OH)ase^−/−^ littermates on the rescue diet. Most parameters were comparable to those of wild‐type mice. These results indicate that 1,25(OH)_2_D_3_ plays a role in preventing bone aging by inactivating p16 signaling through up‐regulating Ezh2.

### 2.7 1,25(OH)_2_D_3_ inhibits cellular senescence and promotes ectopic bone formation of human BM‐MSCs

**Figure 7:**
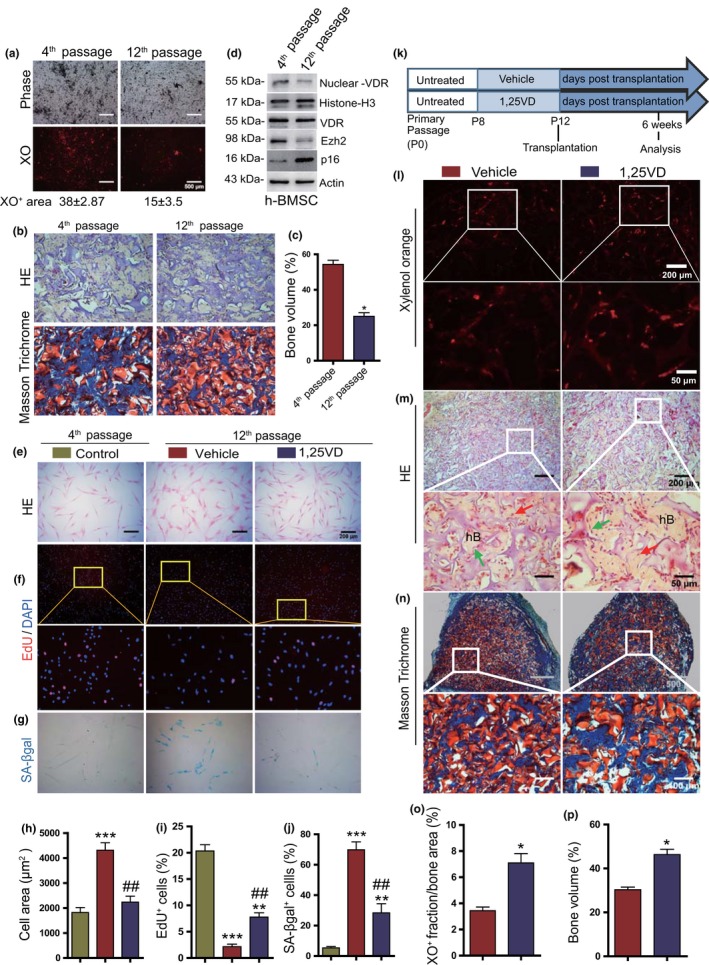
1,25(OH)_2_D_3_ reduces cellular senescence and promotes ectopic bone formation of human BM‐MSCs. (a) Representative micrographs of early (4th) and late (12th) passaged human BM‐MSC cultured for 21 days in osteogenic differentiation medium and stained with xylenol orange (XO) to quantify the percentage of XO^+^ area and (b) were subcutaneously transplanted, After 6 weeks, transplants were harvested and stained with HE (upper part of panel b) or Masson trichrome (bottom part of panel b). (c) The analysis of bone volume based on H＆E staining. (d) Western blot of BM‐MSC extracts from the cultures as (a) for expression of nuclear VDR and total VDR, and for Ezh2 and p16 protein levels. Histone‐H3 was used as nucleoprotein loading control, whereas ß‐actin was used as total protein loading control for Western blots. Representative micrographs of the 4th and 12th passaged BM‐MSCs as (a) stained with (e) H&E, (f) immunocytochemically for EdU, and (g) cytochemically for SA‐β‐gal. Quantification for (h) average cell area, the percentages of (i) EdU^+^, and (j) SA‐β‐gal^+^ cells. (k) Human BM‐MSCs pretreated with vehicle or 1,25(OH)_2_D_3_ and were subcutaneously transplanted into recipient SCID mice. 6 weeks later, the implants were collected and prepared sections were stained (l) with XO, (m) H＆E, and (n) Masson trichrome. Green arrows indicate osteoblasts, and red arrows indicate osteocytes. Bone histomorphometric analysis of (o) mineralization (XO^+^ area, %) and (p) bone volume. *, *p* < .05, **, *p* < .01, ***, *p* < .001, compared to the 4th passaged or vehicle‐treated human BM‐MSCs

Given that 1,25(OH)_2_D_3_ deficiency was associated with the progression of senescence of murine MSCs, we tested whether 1,25(OH)_2_D_3_ treatment could inhibit the senescence and promote in vivo bone formation of human BM‐MSCs. Firstly, we examined whether the reduction of osteogenesis of repeatedly passaged human BM‐MSCs was associated with alterations of VDR‐Ezh2‐p16 signaling. We found that osteogenic potential was significantly reduced in 12th passaged human BM‐MSCs compared with 4th passaged ones as demonstrated in vitro and in vivo (Figure 7a–c). The expression levels of nucleoprotein of VDR and Ezh2 protein were markedly down‐regulated, whereas the expression levels of p16 protein were dramatically up‐regulated in 12th passaged human BM‐MSCs compared with 4th passaged ones (Figure 7d). To determine whether treatment of 1,25(OH)_2_D_3_ could improve the osteogenesis of repeatedly passaged human BM‐MSCs by stimulating their proliferation and inhibiting their senescence, eighth passaged human BM‐MSCs were treated with vehicle or 1,25(OH)_2_D_3_ over 4 passages, and their proliferation and senescence were analyzed. The percentage of EdU^+^ cells was increased significantly, whereas cell size and the percentage of SA‐βgal^+^ cells were reduced dramatically in 1,25(OH)_2_D_3_‐treated 12th passaged human BM‐MSCs, but did not reach the levels of 4^th^ passaged human BM‐MSCs (Figure 7e–j). To further determine whether treatment of 1,25(OH)_2_D_3_ could promote the osteogenesis of repeatedly passaged human BM‐MSCs in vivo, human BM‐MSCs treated with vehicle or 1,25(OH)_2_D_3_ over 4 passages were subcutaneously implanted into recipient NOD‐SCID mice (Figure 7k). In vivo osteoblast differentiation and bone formation were analyzed by staining with XO, H&E, and Masson trichrome. XO^+^ area and bone volume were increased significantly in 1,25(OH)_2_D_3_ pretreated human BM‐MSCs (Figure 7l–p). These findings indicate that 1,25(OH)_2_D_3_ promoted osteogenesis of repeatedly passaged human BM‐MSCs in vitro and in vivo by stimulating their proliferation and inhibiting their senescence mediated though VDR‐Ezh2‐p16 signaling.

## 3 DISCUSSION

We recently reported that 1,25(OH)_2_D_3_ can delay aging in large part by maintaining both calcium/phosphorus homeostasis and redox balance (Chen et al., 2019). The role and mechanism of 1,25(OH)_2_D in maintaining calcium, phosphorus, and skeletal homeostasis have been extensively studied (Fleet, 2017; Veldurthy et al., 2016). The alleged antioxidative effects of vitamin D have been reported in a variety of systems (Bao, Ting, Hsu, & Lee, 2008; Moi, Chan, Asunis, Cao, & Kan, 1994). In a recent study (Chen et al., 2019), we demonstrated that 1,25(OH)_2_D exerts an antioxidant role by transcriptional up‐regulation of Nrf2, mediated via the VDR, subsequently, inhibiting oxidative stress and DNA damage, inactivating p16‐Rb and p53‐p21 signaling pathways, and reducing cell senescence and SASP, thereby resulting in delayed aging (Chen et al., 2019). In this study, we examined whether 1,25(OH)_2_D plays a role in mitigating osteoporosis through its anti‐aging mechanism. To address this question, we first used a 1,25(OH)_2_D‐deficient mouse model, that is, 1α(OH)ase^−/−^ mice, in which serum calcium, phosphorus, and PTH levels were normalized by administering a rescue diet. We found that 1,25(OH)_2_D deficiency accelerated age‐related bone loss by reducing osteoblastic bone formation, increasing osteoclastic bone resorption, activating p16/p19 signaling, and inducing osteocyte senescence and SASP. We then demonstrated that exogenous 1,25(OH)_2_D_3_ supplementation could rescue the bone loss induced by 1,25(OH)_2_D deficiency by reversing these abnormalities.

1,25(OH)_2_D when circulating at high concentrations can increase osteoclastic bone resorption by stimulating the osteoblastic/osteocytic gene expression of RANK ligand, a potent osteoclast regulator. By contrast, deficiency of 1,25(OH)_2_D increased osteoclastic bone resorption by increasing p16 signaling and resorption was reduced when 1,25(OH)_2_D was replaced and when p16 was reduced. Consequently, 1,25(OH)_2_D may regulate osteoclastogenesis and activity via different mechanisms when present in high or low concentrations. Furthermore, we found that natural aging could induce osteocyte senescence and cause an osteogenic defect in BM‐MSCs, whereas exogenous 1,25(OH)_2_D_3_ supplementation could prevent bone loss induced by natural aging through inhibiting p16 signaling, stimulating proliferation, osteoblastic differentiation and osteoblastic bone formation, and inhibiting senescence of osteogenic cells. The results of this study therefore support the concept that 1,25(OH)_2_D plays a role in protecting against osteoporosis through its anti‐aging mechanism.

Accumulating evidence suggests that stem cells play a crucial role in controlling physiological homeostasis, and the rate of stem cell exhaustion is considered one of the hallmarks of aging (Lopez‐Otin et al., 2013). With aging, the alterations of intrinsic properties in MSCs such as senescence, proliferation, and osteogenic/adipogenic differentiation potential may underlie age‐related bone loss (Bergman et al., 1996; Kaneda et al., 2011; Manolagas & Parfitt, 2010; Stenderup, Justesen, Clausen, & Kassem, 2003; Zhou et al., 2008). Previous studies have also demonstrated that VDR deletion leads to premature aging in mice (Keisala et al., 2009) and 1,25(OH)_2_D_3_ has been reported to delay cellular senescence with increased nuclear translocation of VDR in human BM‐MSCs (Klotz et al., 2012). Thus, we asked whether 1,25(OH)_2_D_3_, mediated through VDR, could inhibit osteogenic cellular senescence and stimulate the proliferation and osteogenic differentiation of BM‐MSCs. We found that the nucleoprotein expression levels of VDR were markedly reduced in BM‐MSCs from old mice compared with those from young mice. In addition, VDR nucleoprotein expression levels were also markedly down‐regulated, and the expression levels of p16 protein were dramatically up‐regulated in late passaged human BM‐MSCs compared with early passaged ones. In contrast, 1,25(OH)_2_D_3_ treatment could largely rescue the defects in proliferation and osteogenic differentiation and ectopic bone formation of human BM‐MSCs induced by repeated passaging. Deletion of VDR could inhibit the proliferation and osteogenic differentiation and ectopic bone formation of BM‐MSCs, and increase their oxidative stress and DNA damage and senescence. Our findings therefore indicate that 1,25(OH)_2_D_3_ mediated via VDR stimulates osteogenesis by promoting proliferation and inhibiting oxidative stress, DNA damage, and senescence of BM‐MSCs.

One fundamental aging mechanism that may contribute to multiple age‐related morbidities is cellular senescence (Khosla, Farr, & Kirkland, 2018). There is compelling evidence from preclinical models and supportive human data demonstrating an increase in senescent cells in the bone microenvironment with aging. These cells produce a proinflammatory secretome that leads to increased bone resorption and decreased bone formation, and approaches that either eliminate senescent cells or impair the production of their proinflammatory secretome have been shown to prevent age‐related bone loss in mice (Farr et al., 2017; Sims, 2016). We recently demonstrated that the aging induced by 1,25(OH)_2_D deficiency was accompanied by increased cellular senescence and SASP in multiple organs, whereas exogenous 1,25(OH)_2_D_3_ supplementation could largely rescue the aging phenotypes through reduced cellular senescence and SASP (Chen et al., 2019). Previous reports found a higher proportion of senescent osteocytes and significantly higher expression of multiple SASP markers in old versus young mice (Sims, 2016), suggesting that senescent osteocytes and their SASP may contribute to age‐related bone loss. In our current study, we demonstrated that osteoporosis induced by 1,25(OH)_2_D deficiency as well as by natural aging was accompanied by increased osteocyte senescence and SASP, whereas exogenous 1,25(OH)_2_D_3_ supplementation, by reducing senescent osteocytes and their SASP not only rescued the osteoporotic phenotypes induced by 1,25(OH)_2_D_3_ deficiency, but also largely rescued the osteoporotic phenotypes induced by natural aging.

Whatever the upstream trigger is, the effectors of cellular senescence are molecules governing cell cycle progression such as p16 and p53. The two pathways both act in a concerted way and independently, and different stimuli may activate one pathway, the other, or both (Campisi & d'Adda di Fagagna, 2007). We recently reported that p16 deletion or p53 haploinsufficiency can partly prevent aging resulting from 1,25(OH)_2_D_3_ deficiency by enhancing cell proliferative ability and reducing cell senescence and SASP (Chen et al., 2019). In the current study, we found that 1,25(OH)_2_D_3_ deficiency could activate p16/p19 signaling, whereas exogenous 1,25(OH)_2_D_3_ supplementation could inactivate p16/p19 signaling in 1,25(OH)_2_D_3_ deficient or naturally aging mice. P16, p19, p21, and p53 all increase in senescent cells and are therefore markers of cell senescence, and our in vivo and in vitro results showed that in 1,25(OH)_2_D_3_ deficiency, not only p16, but also p19/p53/p21 were up‐regulated. However, whether they are all causal of in vivo senescence and organismal aging is unclear. Thus, mutant mice with low levels of the mitotic checkpoint protein BubR1 develop progressive aging phenotypes including a short lifespan, abnormal curvature of the spine, and age‐related muscle atrophy. Inactivation of p16 in these mice, increased the lifespan and reduced cell senescence, demonstrating that p16 is an effector of cellular senescence and aging, In contrast, inactivation of p19, a regulator of p53, accelerated aging in vivo and increased cell senescence, demonstrating that p19 delays aging, at least in this model (Baker et al., 2008). Additionally, p21, a target of p53, is also a senescent marker but appears to maintain the viability of senescent cells (Yosef et al., 2017). We therefore used p16 knockout mice as the most appropriate model to assess effects on aging and cell senescence. We demonstrated that p16 deletion not only prolongs the lifespan, but also rescues the bone aging phenotypes of 1α(OH)ase^−/−^ mice on the rescue diet by stimulating osteoblastic bone formation and inhibiting osteoclastic bone resorption, osteocyte senescence, and SASP. Whether p53 haploinsufficiency can also prevent aging‐related osteoporosis caused by 1,25(OH)_2_D deficiency is under investigation in our laboratory. Interestingly, eliminating a relatively small proportion of senescent cells using a “suicide” transgene, INK‐ATTAC, that permits inducible elimination of p16^Ink4a^‐expressing senescent cells upon administration of a drug (AP20187), extends healthspan and prevents the development of multiple age‐related morbidities in both progeroid and normal chronologically aged mice (Baker et al., 2011, 2016). In old mice with established bone loss, activation of the INK‐ATTAC caspase 8 in senescent cells or treatment with senolytics or the JAKi for 2–4 months resulted in higher bone mass and strength and better bone microarchitecture compared to vehicle‐treated mice (Farr et al., 2017), suggesting that targeting cellular senescence could prevent age‐related bone loss in mice. Our findings indicate that 1,25(OH)_2_D_3_ could prevent age‐related bone loss by targeting the p16 cellular senescence pathway.

The Ink4a/Arf locus, consisting of the genes p16^Ink4a^ and p19^Arf^, is central to the induction of senescence, and this locus is tightly controlled by PRC family members (Aguilo et al., 2011; Dhawan, Tschen, & Bhushan, 2009). Ezh2 is a PRC family member that can increase H3K27me3 along the Ink4A/Arf locus, thus repressing p16 and p19 transcription (Cakouros et al., 2012; Li et al., 2017). In this study, we therefore examined whether 1,25(OH)_2_D_3_ repressed the p16/p19 cellular senescence pathway by up‐regulating Ezh2 at a transcriptional level mediated via VDR. We found that the expression levels of Ezh2 were up‐regulated with enriched H3K27me3 near the transcription start site on its promoter region in 1,25(OH)_2_D_3_‐treated BM‐MSCs, and the expression levels of p16^INK4a^ and p19^ARF^ were down‐regulated. The actions of 1,25(OH)_2_D_3_ were partially blocked following Ezh2 inhibitor treatment. The putative promoter region containing the predicted VDR binding sites of the Ezh2 gene was suggested by bioinformatic analysis, and the ability of VDR to physically bind the Ezh2 promoter was confirmed using ChIP‐qPCR. Furthermore, we demonstrated that the putative promoter region containing the predicted VDR binding sites of the Ezh2 gene is sufficient to promote transcription of Ezh2 in the presence of 1,25(OH)_2_D using luciferase assays. We also found that Ezh2 overexpression in VDR‐deficient BM‐MSCs could down‐regulate the protein expression levels of p16 and inhibit BM‐MSC senescence. Overall, our results suggest that 1,25(OH)_2_D inhibits BM‐MSC senescence by VDR‐mediated transcriptional up‐regulation of Ezh2‐H3K27me3, with subsequent repression of p16.

**Figure 8:**
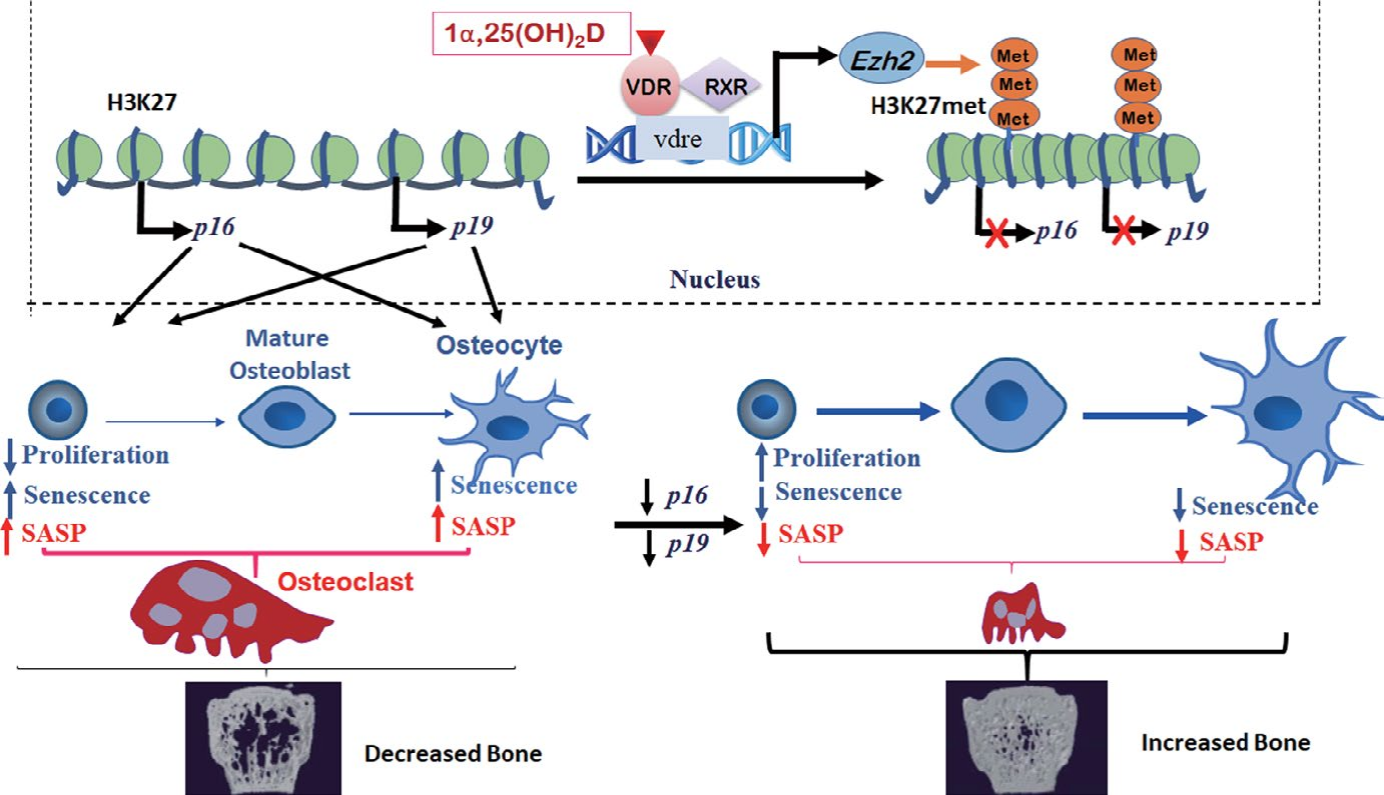
Model of mechanisms used by 1,25(OH)_2_D to protect against age‐related osteoporosis via the VDR‐Ezh2‐p16 signaling axis. 1,25(OH)_2_D binds to the VDR, and the VDR‐RXR heterodimer then binds to the VDRE on *Ezh2* up‐regulating *Ezh2* and increasing H3K27met. This results in repression of *p16/p19* transcription, thus promoting the proliferation of bone marrow MSCs and inhibiting their senescence and SASP production. Senescence and SASP production of osteocytes is also inhibited. As a consequence, osteoblastic bone formation is stimulated, and osteoclastic bone resorption is inhibited. Bone quantity and microarchitecture are thus improved as shown by the μCT images

Renal 1,25(OH)_2_D production is the major source of circulating 1,25(OH)_2_D, and serum 1,25(OH)_2_D levels are inversely related to serum creatinine and glomerular function rate (GFR) in humans. Because many individuals older than 80 have a GFR <50 ml/min, decreased production of 1,25(OH)_2_D in this age group is therefore common (Gallagher, 2013). Our previous results in murine models indicate that the declining 1,25(OH)_2_D levels which occur with aging may in fact contribute to the aging phenotype (Chen et al., 2019). Furthermore, aging‐associated comorbidities including cancer, diabetes, and hypertension have been reported to be influenced by 1,25(OH)_2_D deficiency (Bouillon et al., 2008). The results of the current study now provide a model that suggests that 1,25(OH)_2_D protects against age‐related osteoporosis by up‐regulating Ezh2 via VDR‐mediated transcription, thereby increasing H3K27me3, and repressing p16 transcription, thus promoting the proliferation and osteogenesis of BM‐MSCs, inhibiting their senescence, stimulating osteoblastic bone formation, and inhibiting osteocyte senescence, SASP, and osteoclastic bone resorption (Figure 8). Therefore, by targeting a fundamental aging mechanism, 1,25(OH)_2_D may be an effective agent in the treatment and prevention of age‐related osteoporosis.

## 4 EXPERIMENTAL PROCEDURES

### 4.1 Animals and treatments

The generation and characterization of 1α(OH)ase^−/−^ mice were previously described by Panda et al. (Miao et al., 2001). 1α(OH)ase^−/−^ mice were generated through breeding of heterozygous (1α(OH)ase^+/−^) mice and genotyped by PCR with tail genomic DNA. Gender‐matched 1α(OH)ase^−/−^ and wild‐type (WT) littermates were randomly divided into groups. After weaning, grouped wild‐type and 1α(OH)ase^−/−^ mice were weaned onto one of the following two different regimens: (a) a rescue diet (RD): containing 2.0% calcium, 1.25% phosphorus, and 20% lactose, and (b) a 1,25(OH)_2_D_3_‐supplemented diet (VD), that is, a normal diet plus thrice weekly subcutaneous injections of 1,25(OH)_2_D_3_ at a dose of 1 μg/kg per mouse. Mice were sacrificed at 3, 6, or 12 months of age for skeletal phenotype analysis. To determine the long‐term effect of 1,25(OH)_2_D_3_ supplementation on bone, 12‐month‐old WT mice were injected subcutaneously with 0.1 μg/kg 1,25(OH)_2_D_3_ or vehicle thrice weekly for 6 months and then sacrificed for analysis. Vitamin D receptor knockout mice (VDR^−/−^) (Li et al., 1997) and WT littermates were given RD after weaning, and grouped mice were sacrificed at 3, 6, or 12 months of age for bone marrow mesenchymal stem cell (BM‐MSC) cultures. 1α(OH)ase^+/−^ mice and p16^+/−^ mice were mated to produce offspring heterozygous at both loci (1α(OH)ase^+/−^p16^+/−^), which were then mated to generate 1α(OH)ase^−/−^p16^−/−^ mice. In this study, grouped 6‐month‐old WT, p16^−/−^, 1α(OH)ase^−/−^, and 1α(OH)ase^−/−^p16^−/−^ mice were given the rescue diet after weaning and used for phenotype analysis at 6 months of age and others were maintained until 2 years of age for monitoring lifespan. All animal experiments were performed in compliance with the guidelines approved by the Institutional Animal Care and Use Committee of Nanjing Medical University.

### 4.2 Measurements of serum calcium, phosphorus and 1,25(OH)_2_D_3_

Serum calcium and phosphorus levels were analyzed by an autoanalyzer (Beckman Synchron 67; Beckman Instruments). Serum 1,25(OH)_2_D levels were measured by radioimmunoassay (Diagnostic Products).

### 4.3 Microtomography (μ‐CT)

Vertebrae were removed and examined using μCT as previously described (Sun et al., 2018).

### 4.4 Mechanical testing

Three‐point bending and compression/traction of long bones (femurs and tibias) were performed as described previously (Ren et al., 2011) using a mechanical testing device (Twin Column Table Mounted Testing System; Instron 5565).

### 4.5 Histology and bone histomorphometry

At the time of euthanasia, lumbar vertebrae were dissected free of soft tissue and fixed in PLP fixative buffer for 24 hr at 4°C and washed with PBS for 15 min. For routine histology analysis, decalcified bone in EDTA at 4°C was dehydrated and embedded in paraffin or optimum cutting temperature compound (O.C.T., Tissue‐Tek), after which 5‐μm sections were prepared and bone sections were stained with hematoxylin and eosin (H&E), and for total collagen, beta‐galactosidase (β‐gal) and the osteoclast marker tartrate‐resistant acid phosphatase (TRAP) as previously described (Dimri et al., 1995). For assay of dynamic bone formation, mice were intraperitoneally injected with calcein (10 mg/kg; Sigma) 12 days after which they were given an injection of xylenol orange (XO) (90 mg/kg; Sigma). Nondecalcified bone was embedded in optimum cutting temperature compound (O.C.T), and 7‐μm‐thick sections were obtained using transparent film kindly provided by Prof. Liu Peng. Analyses of dynamic bone formation parameters were performed using standard software kindly provided by Robert J. Van't Hof.

### 4.6 Immunocytochemistry and immunohistochemistry staining

For immunocytochemical staining, cultured cells were incubated with primary antibody against histone H2A on Ser139 (γ‐H2AX; Cell Signaling Technology) overnight at 4℃, followed by using 594‐conjugated goat anti‐rabbit secondary antibody to detect immunoreactivity. For immunohistochemistry staining, dewaxed and rehydrated paraffin‐embedded sections were incubated with 6% hydrogen peroxide to block endogenous peroxidase activity and then washed in PBS (pH 7.6). The slides were then incubated at 4°C overnight with the primary antibodies to β‐gal (Abcam), p16 (Abcam), IL‐6 (Santa Cluz), and Mmp3 (Proteintech). After rinsing with PBS for 15 min, tissues were incubated with secondary antibody (biotinylated goat anti‐rabbit IgG and goat anti‐mouse IgG; Sigma). Sections were then washed and incubated with the Vectastain Elite ABC reagent (Vector Laboratories) for 30 min. Staining was done using 3,3‐diaminobenzidine (2.5 mg/ml) followed by counterstaining with Mayer's hematoxylin. For 5‐ethynyl‐2′‐deoxyuridine (EdU) assay to detect cell proliferation, cultured BMSCs were incubated with EdU for 2 hr, after which EdU staining was performed using Apollo 567 Stain Kit (Ribo‐Bio) according to the manufacturer's instructions. Senescent cells were detected using senescence β‐Galactosidase Staining Kit (Cell Signaling Technology) according to the manufacturer's instructions.

### 4.7 Western blot

Whole‐cell or nuclear lysates were extracted for loading into 10% SDS‐PAGE gels, and immunoblotting was performed as previously described (Miao et al., 2008). Primary antibodies against Ezh2 (Proteintech), p16 (Santa Cruz), p53 (Cell Signaling Technology), p21 (Santa Cruz), p19 (Cell Signaling Technology), VDR (Proteintech), Histone‐H3 (Cell Signaling Technology), and Actin (Cell signaling technology) were used for immunoblotting. Immunoreactive bands were visualized with enhanced chemiluminescence (ECL) (Bio‐Rad) and analyzed by ImageJ.

### 4.8 Cell cultures and lentiviral infection

Primary mouse BM‐MSCs were isolated from grouped WT and VDR^−/−^ mice or from aged WT mice treated with vehicle or 1,25(OH)_2_D_3_, and cultured as described previously (Miao et al., 2001). After obtaining detailed informed consent, primary hMSCs were isolated from bone marrow aspirates during hip replacement surgery for hip osteoarthritis treatment from five male and seven female patients according to procedures approved by the Human Subjects Institutional Review Board of Nanjing Medical University. Patients with hyperparathyroidism, hyperthyroidism, gastrointestinal and nutritional diseases, chronic kidney disease, and long‐term use of glucocorticoids were excluded from this study. BM‐MSCs were cultured in α‐modified Eagle's MEM (Life Technologies) supplemented with 10% FBS (Excell) and 1% penicillin/streptomycin (Life Technologies) at a cell density of 10 × 10^6^/ml. On day 3, nonadherent cells were removed using medium change and fresh medium was replaced twice per week. For osteogenic differentiation, cells were maintained in osteogenic medium (supplemented with 100 nM dexamethasone, 10 mM β‐glycerophosphate, and 50 μg/ml ascorbic acid) for 21 days. Staining for alkaline phosphatase (Alp)‐positive colonies (CFU‐f) and xylenol orange (XO) (for mineralized nodule formation) (Wang, Liu, Buhl, & Rowe, 2005) was performed at 7 and 21 days of culture, respectively. ImageJ software was used to determine areas of Alp and XO staining. For lentiviral infection, viral packaging was performed as described previously (Chen et al., 2018). Briefly, HEK 293T cells were co‐transfected with lentiviral plasmids (pHAGE‐CMV‐MCS‐IZsGreen), packaging vector psPAX2, and envelope vector pMD2.G by using Lipofectamine 2000 (Invitrogen) according to the manufacturer's instructions. Forty‐eight hours after transfection, the supernatants containing lentivirus were harvested for infection in the presence of polybrene (8 µg/ml; Sigma‐Aldrich) for 12 hr. The infection efficiency was determined and showed that almost all BM‐MSCs were infected (GFP^+^). The passage 4 BM‐MSC transduced with lentivirus‐Ezh2 or lentivirus empty vector was shown as lenti‐Ezh2 and lenti‐control, respectively.

### 4.9 ChIP‐qPCR

Chromatin immunoprecipitation (ChIP) was performed using the ChIP kit (Millipore) as previously described (Chen et al., 2019). Briefly, BM‐MSCs were isolated from WT mice aged 12 months treated with vehicle or 1,25(OH)_2_D_3_ (0.1 μg/kg) for 3 months, and then, cell samples were subjected to immunoprecipitation using either a control IgG or the ChIP‐grade VDR (Abcam, ab3508) and H3K27me3 (Cell Signaling Technology) antibody. The coprecipitated chromatin was determined by qPCR for the enrichment of promoter DNA using primers for p16^INK4a^, p19^ARF^, Ezh2, and Gapdh shown in Table S1.

### 4.10 Luciferase reporter assay

To generate the Ezh2 promoter‐activated luciferase reporter, −1702 to +52 bp of Ezh2 promoter was cloned into PGL3 basic. Luciferase reporter assay was performed as previously described (Chen et al., 2019). Briefly, BM‐MSCs in 12‐well plates were transfected with Ezh2‐promoter or Ezh2‐promoter mutant luciferase reporter plasmid. 12 hr later, the medium was changed and the indicated concentration of vehicle or 1,25(OH)_2_D_3_ was added. After another 36 hr, luciferase activity was measured using the Dual‐Luciferase Assay Kit (Promega). pRL‐TK was co‐transfected to normalize transfection efficiency. Each experiment was performed in triplicate and repeated at least three times.

### 4.11 Ectopic bone formation assay

Mouse BM‐MSCs were cultured to 4th passage, after which they were treated with 1,25(OH)_2_D_3_ or vehicle for 72 hr before transplantation. The 8th to 12th passaged human BM‐MSCs were cultured in the presence or absence of 1,25(OH)_2_D_3_ prior to transplantation. The ectopic bone formation model was described previously (Yin et al., 2009). Briefly, the gelfoam used is the sponge mixed with 100% gelatin, which is isolated from pigskin. The gelfoam was then cut into small pieces (5 mm diameter × 5 mm height) for loading the cells at a total of 2 × 10^6^ mouse BM‐MSCs or 1 × 10^6^ human BM‐MSCs. Cells were resuspended in 40 μl α‐MEM and transplanted into the dorsal subcutaneous tissue of 5‐week‐old NOD‐SCID mice. Mice were sacrificed at 6 weeks after transplantation, and implants were collected and processed for staining with H&E and Masson trichrome.

### 4.12 RNA isolation and real‐time RT–PCR

Total RNA was extracted from cultured MSCs and lumbar vertebrae using TRIzol reagent (Invitrogen) according to the manufacturer's instructions. Complementary DNA (cDNA) was synthesized using Synthesis SuperMix (Invitrogen). The real‐time RT–PCR was carried out using an Agilent Real‐time System. Gapdh was amplified at the same time to normalize gene expression. Groups at least six mice were examined, and each experiment was repeated three times to determine relative gene expression differences. The PCR primer sequences used in this study are shown in the Table S2.

### 4.13 Statistical analysis

All analyses were performed using SPSS software (Version 16.0; SPSS Inc.). Measured data were described as mean ± *SEM* fold change over control and analyzed by Student's *t* test, one‐way, or two‐way ANOVA to compare differences between groups. Qualitative data were described as percentages and analyzed using a chi‐square test as indicated. *p* Values were two‐sided, and *p* < .05 was considered statistically significant.

## CONFLICT OF INTEREST

None declared.

## AUTHOR CONTRIBUTIONS

D.M. and D.G. conceived the project. R.Y. and J.C. performed most of the experiments, analyzed, and compiled the data. J.Z., R.Q., R.W., Y.Q., and Z.M. helped with experiments. R.Y., D.M., and D.G. participated in writing or editing the paper.

## Data Availability

The data that support the findings of this study are openly available in https://doi.org/10.1111/acel.12951.
