# Supplementary figures and images for: 1,25‐Dihydroxyvitamin D protects against age‐related osteoporosis by a novel VDR‐Ezh2‐p16 signal axis
Source: Aging Cell. 2019 Dec 26;19(2):e13095. doi: 10.1111/acel.13095 (PMC6996957; doi:10.1111/acel.13095)

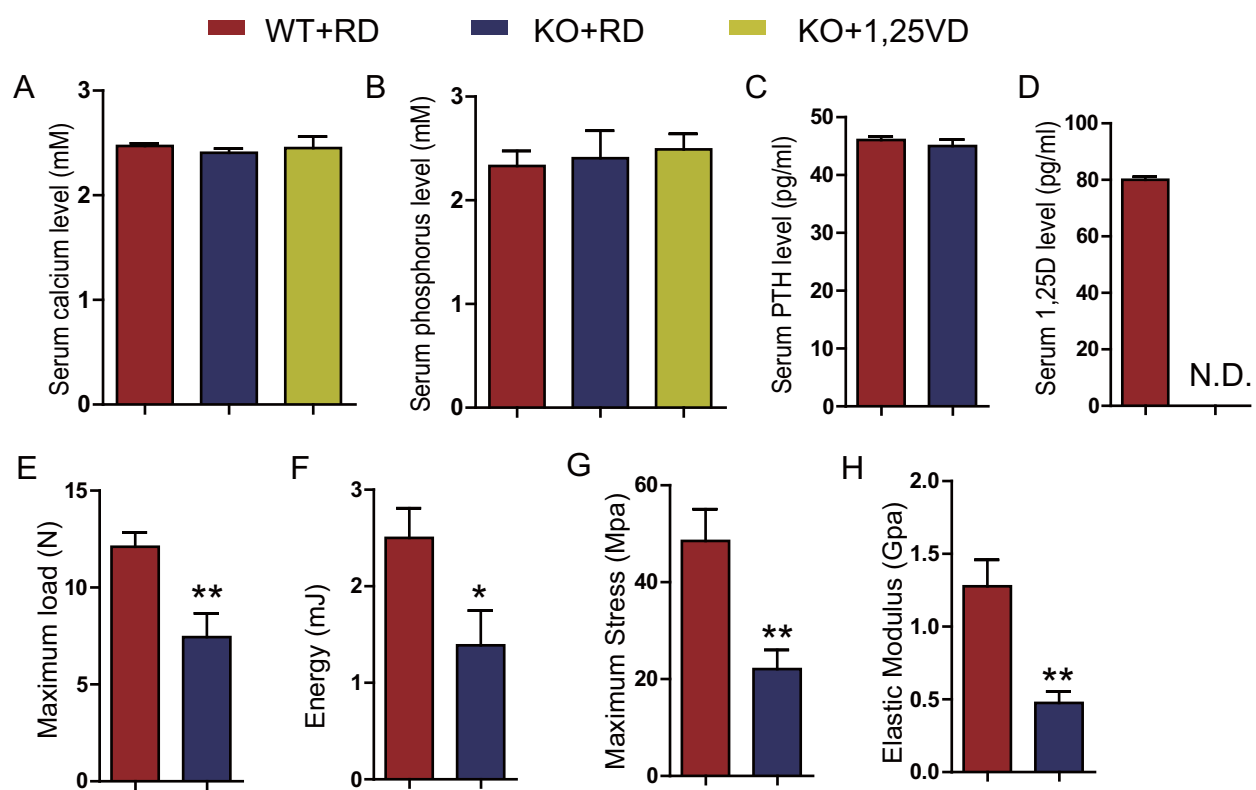

Supplemental Fig.1

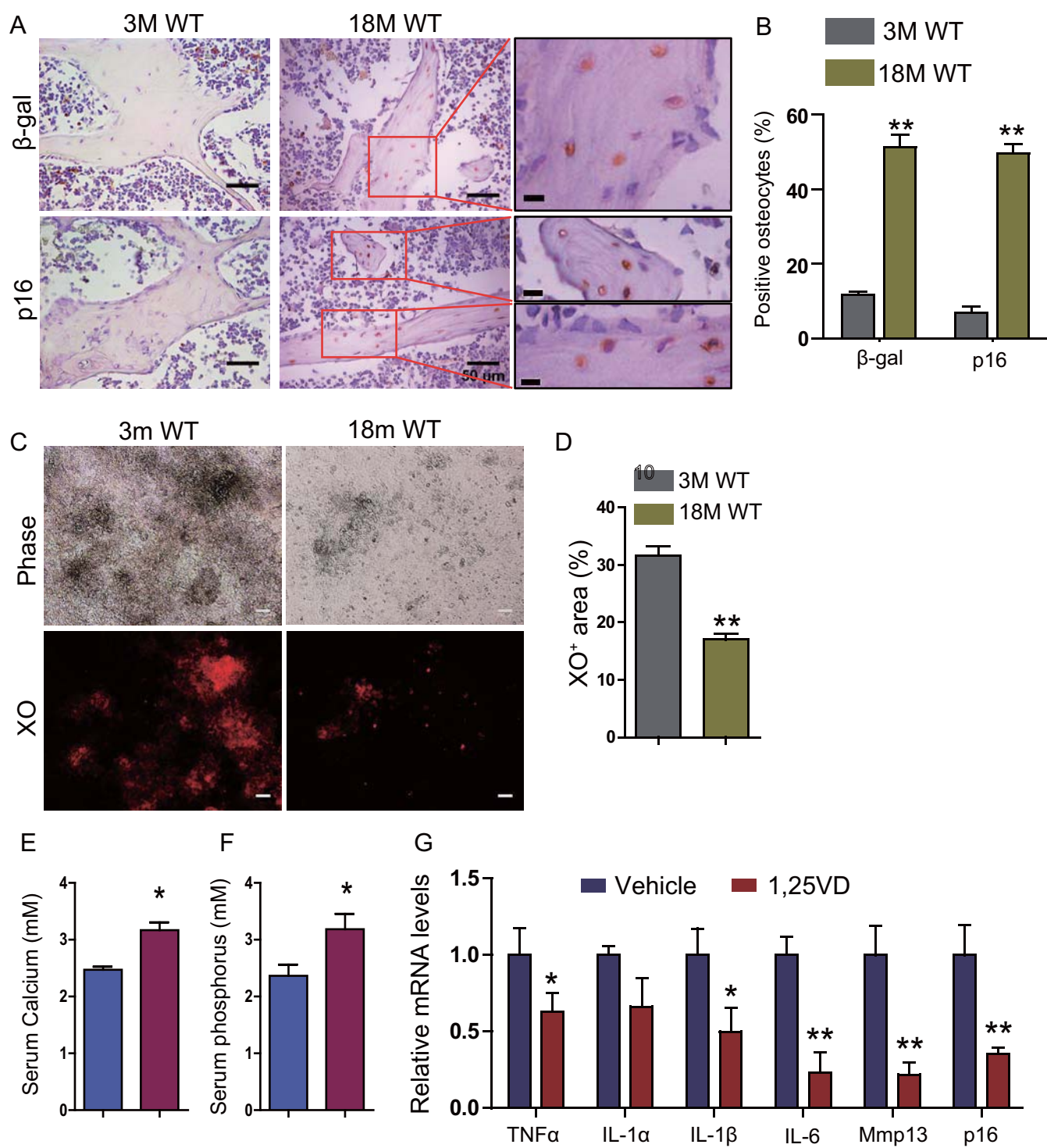

Supplemental Fig.2

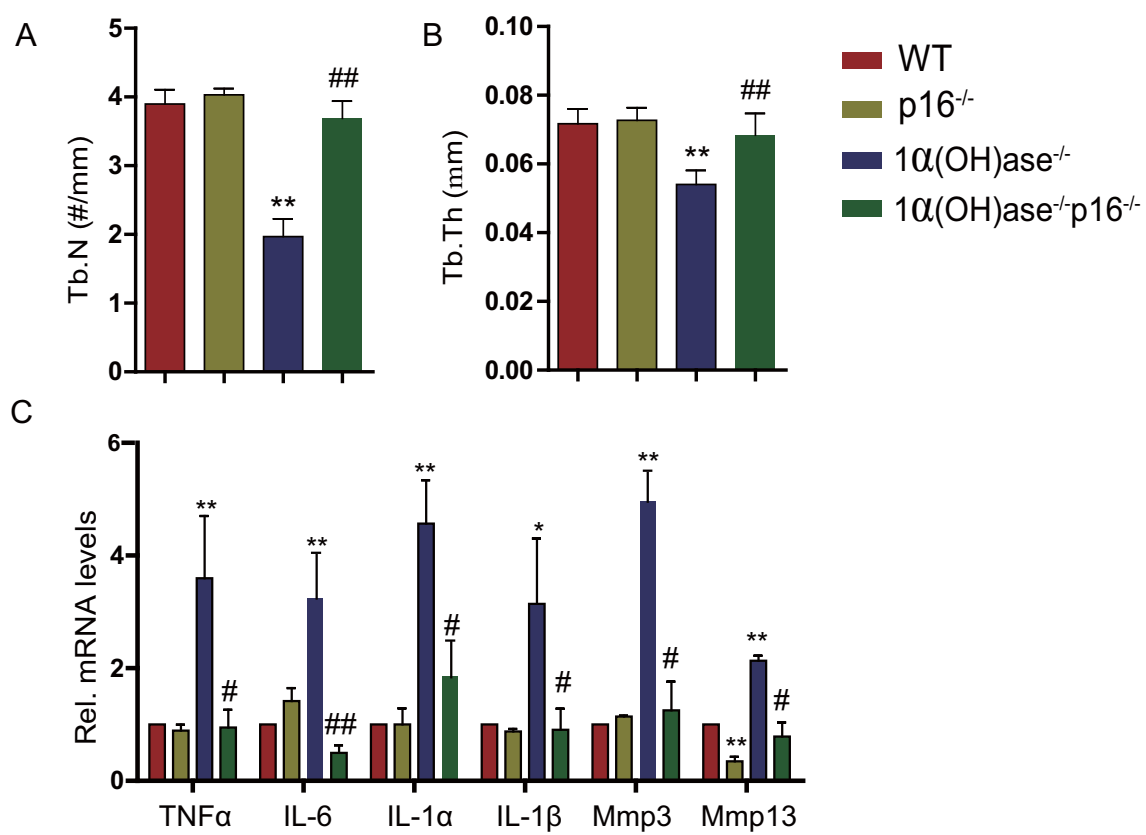

Supplemental Fig.3

Supplement: Supplementary file 1 [file ACEL-19-e13095-s001.pdf]
