# Supplementary material for: 1,25‐Dihydroxyvitamin D protects against age‐related osteoporosis by a novel VDR‐Ezh2‐p16 signal axis
Source: Aging Cell. 2019 Dec 26;19(2):e13095. doi: 10.1111/acel.13095 (PMC6996957; doi:10.1111/acel.13095)
Supplement: Supplementary file 2 [file ACEL-19-e13095-s002.docx]

**Supplementary Table 1. Primers used for quantitative real-time PCR**

| Primers | Forward | Reverse |
| --- | --- | --- |
| TNFα | CCCTCACACTCAGATCATCTTCT | GCTACGACGTGGGCTACAG |
| IL-6 | TAGTCCTTCCTACCCCAATTTCC | TTGGTCCTTAGCCACTCCTTC |
| IL-1α | GCACCTTACACCTACCAGAGT | AAACTTCTGCCTGACGAGCTT |
| IL-1β | GCAACTGTTCCTGAACTCAACT | ATCTTTTGGGGTCCGTCAACT |
| Mmp3 | ACATGGAGACTTTGTCCCTTTTG | TTGGCTGAGTGGTAGAGTCCC |
| Mmp13 | CTTCTTCTTGTTGAGCTGGACTC | CTGTGGAGGTCACTGTAGACT |
| P16^INK4a^ | GAAAGAGTTCGGGGCGTTG | GAGAGCCATCTGGAGCAGCAT |
| P19^ARF^ | CGCAGGTTCTTGGTCACTGT | TGTTCACGAAAGCCAGAGCG |
| P21^CIP1^ | CCTGGTGATGTCCGACCTG | CCATGAGCGCATCGCAATC |
| Ezh1 | CCAGACTGCCAGAATCGCTTT | CAGGTGCTTTTTGAGGCCA |
| Ezh2 | AGTGACTTGGATTTTCCAGCAC | AATTCTGTTGTAAGGGCGACC |
| Gapdh | CCACCCAGAAGACTGTGGAT | GGATGCAGGGATGATGTTCT |

**Supplementary Table 2. Primers used for ChIP-qPCR**

| Primers | Forward | Reverse |
| --- | --- | --- |
| P16^INK4a^-1 | TCCGATCCTTTAGCGCTGTT | CCCGGACTACAGAAGAGATG |
| P16^INK4a^-2 | AGGGGTGTTCAATTCATGCTAT | ACACTCTGCTCCTGACCTGG |
| P16^INK4a^-3 | GGAGCCACCCATTAAACTAACT | CAAAAATAAGACACTGAAAACTCG |
| P19^ARF^-1 | AGGTGCCTCAACGCCGAAG | CTGGTCCAGGATTCCGGTGCGG |
| P19^ARF^-2 | ATGGGCAACGTTCACGTAGCAGC | AGCGGTACACAAAGACCACCCA |
| Ezh2 | TTAGAGTGGCTTACAGGC | ACATCTTCTGGCTTCCTT |
| Gapdh | CCACCCAGAAGACTGTGGAT | GGATGCAGGGATGATGTTCT |
